# The use of a simple anal sling in the management of anal incontinence

**DOI:** 10.1093/gastro/gou012

**Published:** 2014-03-17

**Authors:** José Manuel Devesa, Rosana Vicente

**Affiliations:** ^1^Colorectal Unit, University Hospital Ramón y Cajal, Madrid, Spain and ^2^Colorectal Unit, Hospital Ruber Internacional, Madrid, Spain

**Keywords:** anal incontinence, peri-anal sling, Thiersch

## Abstract

**Background.** Many patients presenting with anal incontinence (AI) are frail, with attendant comorbidities precluding the use of complicated, expensive reconstructive techniques. In these cases, revisiting a simple approach—designed to provide some sort of effective barrier to stool—is worthwhile where the options include a customized peri-anal sling or the use of an anal plug.

**Methods.** Analysis of an unselected cohort of 33 patients (mean age 54 years; range 27–86 years) with AI is presented, these patients having undergone insertion of an elastic band peri-anal sling between December 2004 and December 2009. Pre- and post-operative assessment included the Jorge-Wexner score of incontinence, anorectal manometry and the Rockwood Fecal Incontinence Quality of Life (FIQoL) score.

**Results.** The follow-up period ranged from 50 to 108 months with a mean of 65 months. Early post-operative complications included spontaneous disruption of the sling at the fourth and seventh post-operative day in two patients and local infection in a further two cases. Late complications included skin erosion in two patients, (one occurring 3 years post-operatively) and breakage of the sling in a further seven patients. Explantation was performed in 13 cases, and re-implantation in 10 patients. No differences were noted in resting or squeeze manometry, with significant improvement in the Jorge-Wexner scores in 32 cases and in all of the four Rockwood quality of life scales.

**Conclusions.** An anal sling is an effective and simple surgical option for the management of selected cases presenting with anal incontinence. Longer-term data are awaited comparing this technique with other standard surgical alternatives.

## INTRODUCTION

There is a general consensus that the first-line treatment of non-severe sphincter defects presenting with anal incontinence (AI) that do not respond to conservative therapies is a surgical repair. The details of this approach are covered elsewhere in this Special Edition. The reported degree of success with this approach varies widely, according to well-known factors that include the number of previous attempts and the presence of an attendant pudendal neuropathy [1, 2]. Surgical decisions in patients who fail a sphincter repair—or in the wider population who suffer minor degrees of incontinence without demonstrable sphincter defects—may include complex, expensive procedures that often have a high rate of revision and which may not be suitable or available for high-risk, frail, immobile or elderly patients. Further, in some patients with sphincter atresia—or when direct repair is not feasible or has already failed—recreation of a neosphincter becomes necessary. Here, there is extensive literature concerning the use of muscle transpositions, which may either be stimulated (dynamic) or unstimulated (adynamic), where the principal muscle used is the gracilis. The alternative, in selected cases, is the implantation of an artificial bowel sphincter, which has been translated from urology for the management of severe urge urinary incontinence. Although these complex, specialized procedures have proved successful in a high percentage of patients, they are technically demanding, with a high attendant morbidity and cost and a considerable requirement for correctional surgery. These limitations have resulted in only a few centres regularly performing these operations, with limited access for this worldwide problem [3, 4].

In 1891, Thiersch successfully described a simple method for the treatment of rectal prolapse by encircling the anus with a silver wire [5]. Despite the initially encouraging, good functional results, the technique was temporarily abandoned in view of its high medium- or long-term recurrence rate, where there was frequent anal soiling and problems with wire erosion and also faecal impaction [6]. Despite the initially encouraging results from this easy and low-cost procedure, it has not gained popularity—probably consequent upon frequent breakage of the sling and episodes of faecal impaction [7]—where it has been somewhat abandoned in favour of more sophisticated procedures. The successful peri-anal implantation of an artificial anal sphincter to treat patients with faecal incontinence, though associated with a high incidence of local complications necessitating device explantation, stimulated our group to reconsider the development of a simple mechanical barrier system for implantation around the anus in those cases where there was inherent sphincter deficiency or where other comorbidity factors precluded the use of more complicated techniques. We present our initial experience with the use of a customized elastic sling placed around the anus, designed to prevent gross faecal escape and to improve continence-related quality of life.

## MATERIALS AND METHODS

The device used is a simple elastic band (Jackson-Pratt™ drain) consisting of biocompatible radiopaque silicone, widely used in general surgery. Institutional Board Review approval was obtained for clinical use of the device and informed consent was provided by all patients or their carers, which addressed the experimental nature of the treatment. Between December 2004 and December 2009, anal encirclement was performed in 33 patients with AI. The procedure was used in those who had largely failed prior surgical approaches and who had also failed a trial of conservative and pharmacological therapies. The cohort included patients with daily soiling following low- or ultra-low colorectal anastomosis, after a range of anorectal procedures (most notably after fistula surgery), in cases of anal incontinence coincident with full-thickness rectal prolapse, after failed sphincter repair and in specialized cases following a failed gluteus transposition, a poor outcome after artificial sphincter implantation, or a negative test with sacral nerve stimulation. Pre- and post-operative assessment included the Jorge-Wexner score of incontinence (0 = normal, 20 = complete incontinence) [8], anorectal manometry and the Rockwood Faecal Incontinence Quality of Life (FIQoL) score [9]. Each case served as his or her own control, with data being recorded by an independent trial manager who was not involved in patient care.

### Operative technique

A liquid diet and mechanical cleansing of the rectum was prescribed the day before surgery, with routine peri-procedural systemic antibiotic prophylaxis using a single dose of teicoplanin, (anti-aerobic) and piperacillin-tazobactam (anti-anaerobic) as one dose pre-operatively and two doses post-operatively). The type of anaesthesia (general, regional and local anaesthesia with sedation) varied in accordance with the pre-operative American Society of Anesthesiologists (ASA) status and attendant comorbidity. Subcutaneous tissue around the anus was infiltrated with local anaesthesia and 1/300.000 epinephrine, after which 4–5 minimal incisions were made around the anus, avoiding sites of scar tissue. These incisions were deepened into the ischiorectal fossa and joined subcutaneously with forceps so as to encircle the anus, with care taken to avoid inadvertent entry into the rectum or vagina. The sling was passed along these incisions until both ends met outside the same incision, and the sling was tightened against a finger inserted into the rectum with a clamp placed to maintain the selected tension on the sling at the desired degree of anal tightness. After transection of the sling 2 cm above the tightening clamp, the edges of the sling were sutured to one another with 2/0 Prolene and the wounds were irrigated with a topical gentamicin solution, with the skin being closed with non-absorbable 3/0 monofilament sutures, which were left in place for two weeks. The operative time was <60 minutes in all cases. Post-operatively, patients were provided with a regular diet, 15 patients being discharged on the day of surgery.

## RESULTS

Surgery was performed in 20 women and 13 men (mean age 54 years; range: 27–86 years). Patient characteristics are shown in Table 1. Early post-operative complications included a spontaneous disruption of the sling at the fourth and seventh post-operative days in two patients and local infection in a further two cases. Late complications included skin erosion in two patients, (one occurring three years post-operatively) and breakage of the sling in a further seven patients (Table 2). Sling removal, when necessary, was simply performed under local anaesthesia through the point of sepsis or erosion, with repeat implantation in some cases of erosion. Synchronous or delayed re-insertion of the sling was performed in ten patients with a further explantation in three patients.

**Table 1.** Causes of incontinence

Cause	Patient number (*n* = 33)
Iatrogenic	5 (fistula 4, fissure 1)
Obstetric	5 (failed implant of Acticon® 1)
Idiopathic	5 (failed gluteoplasty 1, negative SNM test 1)
Restorative proctectomy	5 (ileoanal anastomosis 3, coloanal anastomosis 1, coloperineal anastomosis 1)
Rectal prolapse	4
Congenital	4 (failed gluteoplasty 1, failed implant of Acticon® 1)
Neuropathic	3 (failed implant of Acticon® 1, negative SNM test 1)
Mixed	1 (failed gluteoplasty)
Trauma	1

SNM = sacral neuromodulation; Acticon® = artificial anal sphincter.

**Table 2.** Complications after the anal sling procedure

Type	Patient number (%)
Early	
Breakage of the sling	2 (6.1)
Infection	2 (6.1)
Late	
Erosion	2 (6.1)
Late breakage	7 (21.2)
Explantation	13 (39.4)
Re-implantation	10 (30.3)

The follow-up period ranged from 50 to 108 months, with a mean of 65 months. No significant differences were noted between pre- and post-operative resting or squeeze manometric values (Table 3) with, in 32 cases, significant improvement in the functional (Jorge-Wexner) scores and in all of the four Rockwood quality-of-life scales (lifestyle, depression, coping behaviour and embarrassment). One patient presenting with incontinence and a full-thickness rectal prolapse died three years later from an unrelated cause, whilst no patient suffered recurrent rectal prolapse on follow-up (range: 5–46 months).

**Table 3.** Comparison between pre- and post-operative scores

	Pre- operation	Post- operation	*P* value
Mean resting anal pressure, mm Hg; (range)	43 (7–88)	53 (1–90)	NS
Mean anal squeeze pressure, mm Hg; (range)	81 (8–186)	81 (1–227)	NS
Mean Jorge-Wexner score [6] (on a scale of 0–20)	15 ± 5	7 ± 4	<0.001
FIQL^a^ domains			
Lifestyle	2.3 ± 1.0	3.8 ± 0.2	0.038
Depression	1.6 ± 0.4	3.2 ± 0.5	0.007
Coping behaviour	2.3 ± 0.9	3.7 ± 0.4	0.023
Embarrassment	1.6 ± 0.5	3.6 ± 0.5	<0.001

NS = not significant.

^a^FIQL Rockwood Score [7].

## DISCUSSION

The surgical management of severe faecal incontinence that has developed following a low restorative proctectomy (often with attendant irradiation), or that recurs after a sphincter repair or following a more complex procedure—such as a graciloplasty, a gluteoplasty or implantation of an artificial anal sphincter—is challenging. In many cases, the loss of sphincter mass and the presence of prior infection precludes more complex revisional peri-anal surgery, leaving the options limited to performance of an antegrade colonic irrigation system or a permanent colostomy. We resurrected the idea of the simple implantation of a mechanical barrier to support the sphincter (initially used for the management of full-thickness rectal prolapse, with or without faecal incontinence) partly because the more advanced reconstructive procedures require specialized expertise and themselves have a high complication rate [10, 11]. The effect of these more complex, novel reconstructive techniques is compounded by the high cost that is often incurred by the patient, restricting their use to only a few centres worldwide [12]. It is accepted that the results of this simple procedure are, at best, only average where it has been used in a group of end-stage AI patients who have frequently failed other procedures and where a stoma was often the only other viable option. However, the ease of the procedure and its revision (in selected patients) makes it a practical alternative when compared with other, more complicated, procedures that have equivalent complication and explantation rates. Future developments of the technique, with more secure device fixation and more objective intra-operative assessment of the tightness of anal closure, may lead to improved results in prospective studies.

The concept of rectal encirclement, initially used for selected cases of rectal prolapse, has a long history with relatively poor results, largely because of sling breakage and recurrent prolapse, as well as because of troublesome faecal impaction. This older concept was resurrected for use in frail, elderly comorbid patients by Lomas and Coperman [6], who reported excellent results with few complications in a selected cohort of 47 patients, where the main reported technical modifications concerned the shape and type of material used for encircling the anus. This has historically included fascia, tendon, silk, Teflon® tubes, nylon and polyester tapes, Marlex® and silastic mesh and silicone. The data concerning sling use specifically for AI management are limited, where Horn *et al.* published a series of 14 patients in whom a Dacron®-impregnated Silastic® mesh was used with good or excellent results in eight cases [13], and where Poole and colleagues reported continence improvement in six out of nine cases presenting with rectal prolapse, using the same type of mesh [14]. In this regard, Larach *et al.* showed the satisfactory outcome of a modified Thiersch procedure using a Silastic® mesh implant, and proposed this procedure as a simple solution for faecal incontinence specifically coincident with severe prolapse [15].

Our series shows that anal encirclement with the appropriate sling might achieve good or excellent results in a high proportion of selected—often frail—patients with variable degrees of anal incontinence, although we experienced the technical problem of early and late breakage, with a moderately high need for explantation because of this problem and because of erosion. Patients could however be satisfactorily re-implanted, although longer-term data in a larger number of patients is still required. The procedure has the added benefits of being technically simple and easily taught, and can be performed in a general surgical setting without the need for specialist coloprotcological facilities. It can be performed even under local anaesthesia and, in many patients, as an ambulatory procedure and was not associated with post-operative faecal impaction.

It is accepted that this preliminary Phase I data has the limitation of not being conducted as a prospective, randomized, comparative study between techniques, aiming in its initial stage to assess the feasibility of device implantation, its safety profile and preliminary functional outcome. Future studies will retrospectively compare this ongoing data accumulation with historical controls undergoing other continence-enhancing procedures along with the performance of a prospective, randomized controlled trial assessing use of the sling in comparison with other techniques. Further development of this simple but effective device will focus on technical modifications designed to prevent early and late sling breakage and erosion.

**Conflict of interest:** none declared.
